# Distribution and laterality of concha bullosa in patients with different cranial skeletal types: a retrospective analysis among cases with concha bullosa

**DOI:** 10.1186/s40902-025-00463-y

**Published:** 2025-05-08

**Authors:** Farhad Ghorbani, Ali Modaberi, Nasim Morshedian, Ali Gorgin, Maryam Paknahad

**Affiliations:** 1 Shiraz university of medical sciences, Shiraz, Islamic Republic of Iran; 2 School of Dentistry, Shiraz University of Medical Sciences, Shiraz, Islamic Republic of Iran; 3 Oral and dental disease research center, oral and maxillofacial radiology department, Shiraz, Islamic Republic of Iran

**Keywords:** Concha Bullosa, Cranial Morphology, Paranasal Sinuses, Computed Tomography, Nasal Obstruction

## Abstract

**Objective:**

Concha bullosa, a common anatomical variation characterized by air-filled cavities in the turbinate bones, can influence sinonasal function and surgical planning. This study aims to evaluate the distribution, laterality, and cranial skeletal type associations of concha bullosa (CB) among patients with confirmed CB findings on computed tomography (CT) scans.

**Methods:**

A retrospective cohort study was conducted on 774 patients who underwent cranial and facial CT scans between March 2023 and March 2024. Patients were classified into mesocephalic, brachycephalic, and dolichocephalic groups based on the cephalic index. The distribution and laterality of concha bullosa were assessed using CT scans, and statistical analyses were performed using the Chi-square test, with a significance level set at *P* < 0.05.

**Results:**

Among CB-positive patients, left-sided concha bullosa was most common (49.48%), followed by right-sided (31.91%) and bilateral (18.6%) involvement. Mesocephalic individuals constituted the largest proportion of CB-positive cases (55.56%), followed by dolichocephalic (22.86%) and brachycephalic (21.57%) individuals. A significant gender difference was observed in the mesocephalic (*P* = 0.001) and brachycephalic (*P* = 0.013) groups, with males exhibiting a higher prevalence of right-sided and bilateral concha bullosa.

**Conclusion:**

Concha bullosa distribution varies significantly among cranial skeletal types among CB-positive patients, with mesocephalic individuals exhibiting the highest overall prevalence. Our findings underscore the influence of cranial morphology on the presentation of CB. This insight may enhance radiological evaluation and individualized surgical planning in CB-positive patients.

## Introduction

Concha bullosa is one of the most common anatomical variations, characterized by the presence of air-filled cavities within the turbinate bones, most frequently affecting the middle turbinate. It can be unilateral or bilateral [1] and, in some cases, may also involve the inferior turbinate although it is more prevalent in the middle turbinate and may extend to the superior turbinate [2]. Based on the site of pneumatization, concha bullosa can be classified into three types: lamellar, bulbous, and extensive [3]. Various studies have reported a wide range of prevalence for concha bullosa, ranging from 5 to 70% [4], with this variability attributed to factors such as racial differences [5]. While concha bullosa is often asymptomatic and incidentally discovered through computed tomography (CT) imaging, excessive pneumatization of the middle turbinate may lead to nasal obstruction, headaches, septal deviation, and chronic sinusitis [6]. The impact of concha bullosa on the paranasal sinuses is significant as it can contribute to mucus thickening and retention, potentially resulting in sinusitis due to mechanical obstruction of the sinus outflow tract [7]. Among the most common complications associated with concha bullosa are septal deviation and sinusitis [3]. CT scanning is considered the most reliable method for diagnosing concha bullosa as it provides cross-sectional imaging through X-ray technology, enabling the construction of detailed three-dimensional representations of the anatomical structures [8].

Individuals exhibit different cranial skeletal forms based on the cephalic index, which is calculated as the ratio of head width (the distance between the two parietal bones) to head length (the distance from the frontal bone to the occipital bone). According to this index, head shape is classified into three types: mesocephalic, brachycephalic, and dolichocephalic. Mesocephalic individuals have a natural head proportion, with cephalic indices ranging from 76 to 81% in males and 77% to 82% in females. Brachycephalic individuals have a broader and shorter head shape with an increased cephalic index, while dolichocephalic individuals have a longer and narrower head shape with a decreased cephalic index [9].

Recognizing concha bullosa is essential before performing nasal surgeries such as rhinoplasty and maxillofacial trauma surgeries to ensure appropriate preoperative planning. Given the close relationship between craniofacial architecture and paranasal sinus anatomy, cranial skeletal morphology may play a role in predisposing individuals to specific sinonasal variations, including concha bullosa. The cephalic index—a ratio of head width to head length—is commonly used to categorize cranial shapes into mesocephalic, brachycephalic, and dolichocephalic types. These skeletal patterns reflect underlying structural differences that may influence the pneumatization of the nasal turbinates and, consequently, the risk of nasal obstruction or sinus pathology.

Morphological differences in cranial skeletal types, classified by the cephalic index into mesocephalic, brachycephalic, and dolichocephalic categories, may influence the development and expression of sinonasal anatomical variations. Therefore, a thorough understanding of this anatomical variation can aid in the management of associated conditions. However, limited research exists evaluating how these cranial forms correlate with CB characteristics among confirmed cases. This study addresses that gap by analyzing CB distribution and laterality across different cranial types exclusively among patients with CT-confirmed CB.

## Material and methods

### Study design

This retrospective cohort study received ethical approval from institutional Ethics Committee (IR.SUMS.DENTAL.REC.1401.079) and was conducted in accordance with ethical guidelines to ensure patient data confidentiality and privacy.

### Sample size and selection

Among 1646 patients who underwent CT imaging at Shahid Rajaee Hospital between March 2023 and March 2024, 774 (47%) were identified as having at least one form of concha bullosa. Only these 774 CB-positive patients were included in the subsequent analysis of cranial skeletal type and laterality. Therefore, this study analyzes the anatomical distribution of CB solely within the subset of individuals who were CB-positive, rather than assessing population-level prevalence. A census sampling method was employed, ensuring that all eligible cases within the study period were analyzed.

### Inclusion and exclusion criteria

The study included patients aged 18 years or older who underwent cranial and facial CT scans at Shahid Rajaee Hospital and had complete and accessible medical records. Patients were excluded if they had incomplete medical records, congenital craniofacial anomalies, a history of nasal trauma, or prior sinus surgery, malignancy, or acute/chronic rhinosinusitis.

## CT imaging protocol

All cranial and facial CT scans were performed using a Siemens Somatom multi-detector CT scanner. Scanning was done in the axial plane with a slice thickness of 1 mm, pitch of 1.0, and reconstruction interval of 1 mm. High-resolution bone algorithm was used for image reconstruction. Images were obtained without the use of contrast agents. The dataset was reconstructed in axial, coronal, and sagittal planes to allow comprehensive assessment of paranasal sinus anatomy.

### Evaluation method

**Fig. 1:**
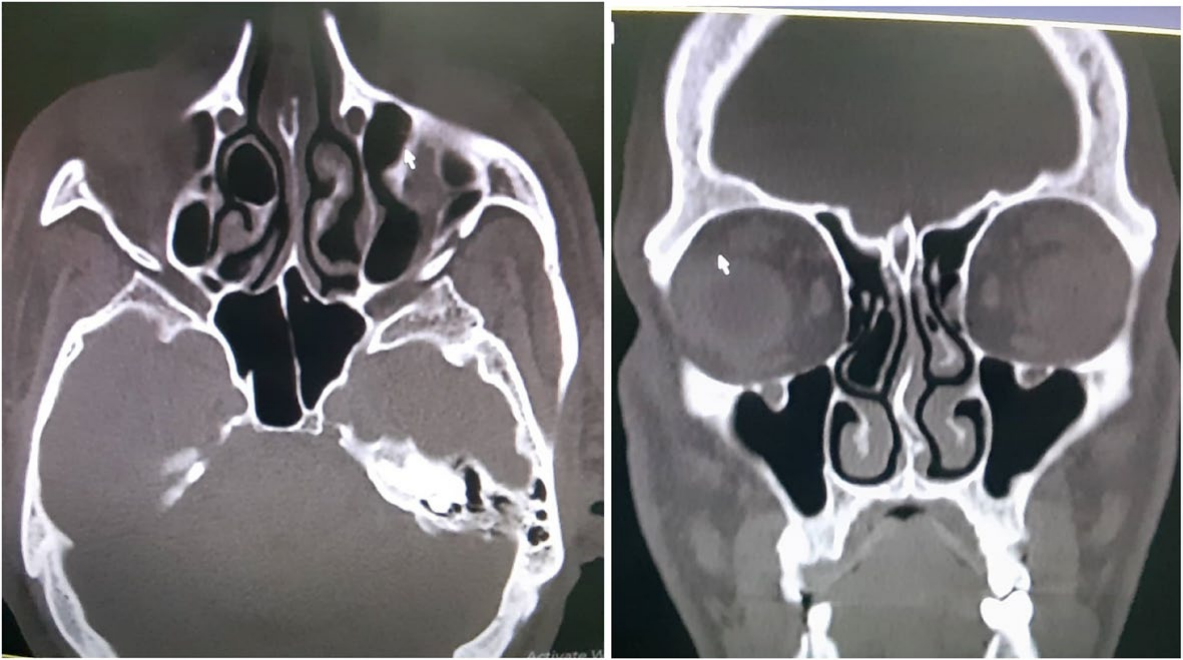
Axial and coronal computed tomography (CT) images showing unilateral concha bullosa involving the right middle turbinate. The pneumatized middle turbinate is clearly outlined, indicating more than 50% vertical height involvement, consistent with the diagnostic threshold for CB

CT images were assessed for the presence and laterality of concha bullosa, defined as pneumatization involving more than 50% of the vertical height of the middle turbinate [10]. Evaluations were conducted using multi-planar reconstruction to improve diagnostic accuracy. Multi-planar CT images were assessed to identify the presence and laterality of CB, as illustrated in Fig. 1. Concha bullosa was classified as unilateral (right or left) or bilateral. Additionally, images were assessed in a standardized viewing environment with consistent window settings optimized for bone structures.

### Observer qualifications and reliability

All CT scans were independently reviewed by two board-certified radiologists (each with more than 5 years of experience in head and neck imaging) and one otolaryngologist. In cases of discrepancy, consensus was reached through discussion. To ensure reliability, a random subset of 50 scans was re-evaluated after a 2-week interval by the same observers to assess intraobserver agreement, with Cohen’s kappa statistics used to quantify consistency.

#### Study measurements

Patients’ medical records were systematically reviewed, and data were extracted daily from the hospital statistics unit, with only complete and eligible records included and entered Microsoft Excel for preliminary processing. The cranial skeletal classification was determined based on the cephalic index, calculated as the ratio of biparietal distance (head width) to head length (distance from the frontal bone to the occipital bone), categorizing patients as mesocephalic (76–81% in males, 77–82% in females), brachycephalic (broader and shorter head with an increased cephalic index), or dolichocephalic (longer and narrower head with a decreased cephalic index), while CT scan findings were used to classify concha bullosa as unilateral (right or left) or bilateral.

#### Primary outcome

The primary outcome of the study was to determine the prevalence of concha bullosa across different cranial skeletal structures. Concha bullosa was diagnosed based on the presence of air-filled cavities in the middle turbinate, as observed in CT scan imaging.

#### Statistical analysis

All data were analyzed using SPSS software (version 24); descriptive statistics (frequency and percentage) were used to summarize categorical variables, while the Chi-square test (χ^2^) was applied to assess the associations between categorical variables, considering a P-value < 0.05 as statistically significant. It is important to note that all statistical proportions reported reflect distributions within the CB-positive population and do not represent incidence or risk factors in the general population. The association between cephalic index and CB laterality should therefore be interpreted as morphological correlation rather than causal inference.

## Results

**Fig. 2:**
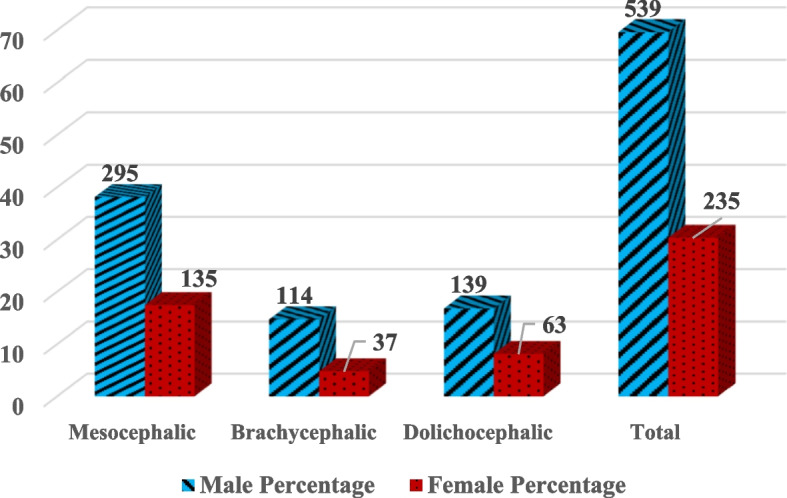
Distribution of cranial skeletal types by gender among CB-positive patients (n = 774). This chart summarizes the relative frequency of mesocephalic, brachycephalic, and dolichocephalic types in male and female participants with confirmed concha bullosa

A total of 774 patients were included in the study, comprising 539 males (69.63%) and 235 females (30.37%). The distribution of cranial skeletal types among the participants was as follows: mesocephalic (55.56%), brachycephalic (21.57%), and dolichocephalic (22.86%). The prevalence of concha bullosa was analyzed based on laterality (right, left, and bilateral) across these skeletal types. The gender distribution within each cranial skeletal type among CB-positive patients is shown in Fig. 2.

A high correlation coefficient of 0.98 was observed between the different measurements, reflecting a strong agreement and high precision in the data. Furthermore, reliability was assessed by re-evaluating 50 randomly selected samples, which yielded a correlation coefficient of 0.97, confirming the reproducibility and consistency of the measurement methods. Statistical tests showed no significant differences in measurement accuracy across different conditions, indicating reliable and repeatable results.

### Prevalence of concha bullosa across cranial skeletal types

The overall prevalence of concha bullosa showed a dominant left-sided occurrence (49.48%), followed by right-sided one (31.91%), while bilateral involvement was the least common (18.6%). The mesocephalic group exhibited the highest prevalence of concha bullosa (55.56% of total cases), with left-sided occurrence being the most frequent (32.04%), followed by the right (17.05%) and bilateral (6.45%) conditions. Among brachycephalic individuals (21.57% of total cases), bilateral concha bullosa was the most prevalent (9.17%), followed by right-sided (7.10%) and left-sided (5.29%). In contrast, dolichocephalic individuals (22.86% of total cases) exhibited a predominant left-sided occurrence (12.14%), followed by right-sided (7.75%), while bilateral involvement was the least common (2.97%) (Table 1). *P*-value refers to comparisons between the cranial skeletal types (mesocephalic, brachycephalic, and dolichocephalic) and the prevalence of concha bullosa by laterality (right, left, and bilateral). The statistical analysis using the Chi-square test revealed a significant association (*P* < 0.001) between cranial skeletal type and the laterality distribution of concha bullosa, indicating that the distribution of right, left, and bilateral cases varies significantly between mesocephalic, brachycephalic, and dolichocephalic types.

**Table 1:** Prevalence of concha bullosa by cranial skeletal type

Cranial Skeletal Type	Right Side	Left Side	Bilateral	Total	*P*-value
Mesocephalic	132 (17.05)	248 (32.04)	50 (6.45)	430 (55.56)	< 0.001*
Brachycephalic	55 (7.10)	41 (5.29)	71 (9.17)	167 (21.57)	
Dolichocephalic	60 (7.75)	94 (12.14)	23 (2.97)	177 (22.86)	
Total	247 (31.91)	383 (49.48)	144 (18.6)	774 (100)	

The values indicate as frequency(percentage)

^*^Indicates as significant *P*-value

### Gender-based distribution of concha bullosa

A statistically significant difference in concha bullosa prevalence was observed between males and females in both mesocephalic (*P* = 0.001) and brachycephalic (*P* = 0.013) groups, while no significant difference was found in the dolichocephalic group (*P* = 0.358) (Table 2).

**Table 2:** Prevalence of Concha Bullosa by Cranial Skeletal Type and Gender

**Cranial Skeletal Type**		**Male** **(*n* = 539)**	**Female** **(*n* = 235)**	***P*-value**
Mesocephalic	*Right Side*	107(36.3)	25(18.5)	0.001*
	*Left Side*	156(52.9)	92(68.1)	
	*Bilateral*	32(10.8)	18(13.3)	
Brachycephalic	*Right Side*	42(32.3)	13(35.1)	0.013*
	*Left Side*	26(20)	15(40.5)	
	*Bilateral*	62(47.7)	9(24.3)	
Dolichocephalic	*Right Side*	36(31.6)	24(38.1)	0.358
	*Left Side*	65(57)	29(46)	
	*Bilateral*	13(11.4)	10(15.9)	

The values indicate as frequency(percentage)

^*^Indicates as significant *P*-value

In the mesocephalic group, right-sided concha bullosa was significantly more common in males (36.3%) compared to females (18.5%) (*P* = 0.001). Conversely, left-sided concha bullosa was more frequent in females (68.1%) than males (52.9%), while bilateral concha bullosa was relatively low in both genders (10.8% in males, 13.3% in females).

Among brachycephalic individuals, bilateral concha bullosa was significantly higher in males (47.7%) compared to females (24.3%) (*P* = 0.013). Similarly, left-sided concha bullosa was more frequent in females (40.5%) than in males (20.0%). However, the right-sided prevalence was comparable between both genders (32.3% in males vs. 35.1% in females).

In the dolichocephalic group, there was no statistically significant gender difference (*P* = 0.358). However, left-sided concha bullosa remained the most prevalent in both males (57.0%) and females (46.0%), followed by right-sided (31.6% in males, 38.1% in females), with bilateral involvement being the least common (11.4% in males, 15.9% in females).

## Discussion

Several previous studies have investigated the prevalence and anatomical variations of concha bullosa; however, few have specifically explored its correlation with cranial skeletal types based on cephalic index. For instance, studies by El-Din et al. [9] and Aktas et al. [11] examined the association between concha bullosa and sinusitis or septal deviation, but did not evaluate cranial morphology as a contributing factor. While prior studies have explored associations between cranial morphology and sinonasal variations, our study contributes additional data by focusing exclusively on CB-positive cases and assessing laterality in detail. By incorporating cephalic index measurements, our findings provide new insights into the potential anatomical predispositions for concha bullosa development and its clinical implications. This study aimed to assess the prevalence of concha bullosa across various cranial skeletal types and its association with gender and laterality. Our findings indicate that concha bullosa is most commonly left-sided (49.48%), followed by right-sided (31.91%), with bilateral involvement being the least common (18.6%). Mesocephalic individuals exhibited the highest prevalence (55.56%), while brachycephalic individuals demonstrated the highest proportion of bilateral concha bullosa (9.17%). These results suggest a potential influence of cranial morphology on the anatomical presentation of concha bullosa, which may have implications for both clinical evaluation and surgical planning.

The prevalence of concha bullosa in this study was 47%, which is considered relatively high and consistent with previous reports in the literature. For instance, a study which analyzed CT scans reported that 67.5% of patients exhibited pneumatization of at least one concha, with 43.2% showing bilateral involvement. This prevalence is higher than in other studies, where concha bullosa varied from 35 to 53% (Smith et al.) [12]. The variation in prevalence rates across studies may be attributed to differences in study populations and diagnostic criteria. In a pediatric population, the prevalence of concha bullosa was found to be 39.8%, with a significant increase in children over four years old. The most common type observed was the lamellar type (Jiang et al.) [13]. This suggests that the development of concha bullosa may continue with age, potentially influencing its prevalence in different age groups.

Consistent with previous studies by Stallman et al. [10] and Smith et al. [12], our data confirm a higher prevalence of CB in mesocephalic individuals, particularly with left-sided dominance. This may relate to their balanced craniofacial architecture, which may permit greater middle turbinate pneumatization.

Our findings revealed that brachycephalic individuals had the highest proportion of bilateral concha bullosa among all cranial types. The broader cranial base of brachycephalic skulls may contribute to more symmetrical turbinate pneumatization, explaining the higher frequency of bilateral CB observed in this group. These results are consistent with previous reports by El-Din et al. [9] and Aktas et al. [11]. supporting the notion that cranial morphology may influence the laterality pattern of CB.

Conversely, our study found that brachycephalic individuals exhibited the highest incidence of bilateral concha bullosa, which is consistent with the findings of El-Din et al. [9] and Aktas et al. [11]. Brachycephalic individuals exhibited the highest frequency of bilateral CB, which may be attributed to the structural symmetry associated with their broader cranial base.

A significant gender-based difference was observed in the prevalence of concha bullosa among mesocephalic and brachycephalic individuals, with males exhibiting a higher prevalence of right-sided concha bullosa and bilateral involvement compared to females. These findings are in agreement with a previous study by Sogono et al. [14], which suggests that hormonal and genetic factors may influence the pneumatization process of the middle turbinate.

Understanding the relationship between cranial skeletal types and concha bullosa may be helpful for improving preoperative planning in rhinologic and maxillofacial surgeries. Given that concha bullosa can contribute to nasal obstruction, septal deviation, and sinusitis, individualized anatomical assessments should be considered before surgical interventions such as functional endoscopic sinus surgery (FESS) and septoplasty. The tendency toward bilateral CB in brachycephalic individuals may complicate nasal airflow and should be accounted for in preoperative evaluation of sinonasal disorders. However, further prospective studies are needed to determine whether cranial skeletal type can reliably inform surgical decision-making or predict treatment outcomes.

Additionally, our findings highlight the importance of considering cranial morphology in radiological evaluations. Radiologists should be aware of the varying prevalence rates of concha bullosa across different skeletal types to improve diagnostic accuracy and facilitate early intervention in symptomatic cases.

While our study provides valuable insights into the relationship between cranial skeletal types and concha bullosa prevalence, certain limitations should be acknowledged. First, this study was conducted at a single center, which may limit the generalizability of our findings to broader populations. Second, the study design was retrospective, and variations in CT imaging techniques and interpretation may have introduced potential biases. Environmental factors (e.g., air pollution, allergens) and genetic predisposition were not considered, though they may influence Concha Bullosa development. Additionally, family history was not assessed, despite potential hereditary links. Variations in CT scan protocols could also impact findings. Additionally, since our analysis included only patients with confirmed concha bullosa, the findings reflect distribution patterns within the CB-positive population rather than the general population. As such, associations with cranial skeletal types observed in this study should not be interpreted as indicative of risk or prevalence trends across the broader population. Including non-CB individuals in future studies would allow for a more comprehensive understanding of potential predisposing craniofacial factors.

Future studies should aim to incorporate larger, multi-center cohorts to validate our findings and explore additional factors influencing concha bullosa development, such as genetic predisposition and environmental influences. Furthermore, longitudinal studies assessing the impact of concha bullosa on nasal airflow dynamics and quality of life in affected individuals could provide further clinical insights.

## Conclusion

This study demonstrates that the prevalence of concha bullosa varies significantly across cranial skeletal types, with mesocephalic individuals exhibiting the highest overall prevalence and brachycephalic individuals showing the highest incidence of bilateral involvement. Left-sided concha bullosa was the most common presentation across all skeletal types. These findings have important clinical implications for the diagnosis and management of sinonasal disorders and emphasize the need for personalized surgical planning based on cranial morphology. These findings also can aid in preoperative planning for sinus surgery and the management of patients with nasal obstruction and chronic sinusitis.

## Data Availability

The data that support the findings of this study are available on request from the corresponding author.

## Abbreviations

CT: Computed tomography
FESS: Functional endoscopic sinus surgery
